# Anticholinergic load and quality of life in Australian residential aged care: a retrospective cohort study

**DOI:** 10.1093/intqhc/mzaf123

**Published:** 2025-12-16

**Authors:** Ying Xu, Ling Li, Nasir Wabe, Magdalena Z Raban, Guogui Huang, Amy D Nguyen, S Sandun Malpriya Silva, Rachel Urwin, Johanna I Westbrook

**Affiliations:** Australian Institute of Health Innovation, Macquarie University, North Ryde, NSW, Australia; Australian Institute of Health Innovation, Macquarie University, North Ryde, NSW, Australia; Australian Institute of Health Innovation, Macquarie University, North Ryde, NSW, Australia; Australian Institute of Health Innovation, Macquarie University, North Ryde, NSW, Australia; Australian Institute of Health Innovation, Macquarie University, North Ryde, NSW, Australia; Australian Institute of Health Innovation, Macquarie University, North Ryde, NSW, Australia; St Vincent’s Clinical Campus, UNSW Sydney, Darlinghurst, NSW, Australia; Australian Institute of Health Innovation, Macquarie University, North Ryde, NSW, Australia; Australian Institute of Health Innovation, Macquarie University, North Ryde, NSW, Australia; Australian Institute of Health Innovation, Macquarie University, North Ryde, NSW, Australia

**Keywords:** anticholinergic load, medication use, wellbeing

## Abstract

**Background:**

The specific impact of anticholinergic load on quality of life is understudied. We aimed to investigate relationships between anticholinergic load and quality of life in residential aged care facilities and differences between residents with and without dementia.

**Methods:**

We conducted a retrospective cohort study of 21 residential aged care facilities in New South Wales, Australia. Residents aged ≥65 years for permanent care. Residents had their quality of life measured using the Quality of Life Aged Care Consumers tool in 2023 at baseline (July–September) and follow-up (October–December, the study outcome). Higher scores indicate better quality of life. Anticholinergic load of administered medications between two quality of life measures was evaluated using five existing scales: Anticholinergic Cognitive Burden, Anticholinergic Drug Scale, Anticholinergic Loading Scale, Anticholinergic Risk Scale, and Clinician-rated Anticholinergic Score. Associations between anticholinergic load from each scale and follow-up quality of life scores were examined using linear regression, controlling for age, sex, baseline quality of life scores, and other potential confounders. Interactions between anticholinergic load and dementia were tested. Analyses were conducted for overall cohort and a subgroup analysis was performed for residents with and without dementia.

**Results:**

A total of 927 residents (69.7% female) were included. One-point higher anticholinergic load measured on each of the Anticholinergic Loading Scale, Anticholinergic Risk Scale, and Clinician-rated Anticholinergic Score, was associated with lower quality of life scores at follow-up: −0.24 (95% confidence interval −0.47, −0.01), −0.26 (95% confidence interval −0.46, −0.05), and −0.25 (95% confidence interval −0.49, −0.01), respectively. Associations did not differ by dementia status. In a subgroup analysis, the associations remained statistically significant in residents without dementia (*n* = 501), but not in those with dementia (*n* = 426).

**Conclusion:**

Our results indicate that, controlling for baseline quality of life, anticholinergic load was associated with lower quality of life at follow-up over a short period (up to 6 months).

## Introduction

There has been increasing awareness given to medication use in Australian residential aged care facilities (RACFs) [1]. As part of a national Quality Indicator program, all government-funded RACFs are required to report two medication management quality measures, in each quarter: the proportions of residents who were prescribed nine or more medications; and residents who received any antipsychotic medications [1]. Among many other medication safety issues not yet covered in the national program is overuse of medications with anticholinergic effects (anticholinergics) [2]. Anticholinergics are widely used for common conditions such as Parkinson’s disease, bradycardia, chronic obstructive pulmonary disease, depression, and urinary incontinence [2]. Yet, many guidelines recommend avoiding anticholinergics in older adults, especially those with dementia [2, 3]. The main concerns are their central nervous system side effects, such as dizziness, drowsiness, confusion, and cognitive decline, and their opposing actions to anticholinesterases, the most used pharmaceutical treatment for Alzheimer’s disease [2, 3]. They also have peripheral side effects, including blurring vision, dry eye and mouth, tachycardia, urinary retention, and constipation [3]. There are cumulative anticholinergic effects from different medications an individual is taking, called anticholinergic load [4].

Quality of life (QoL) refers to how individuals perceive their position in life, considering their environment and goals, expectations, standards, and concerns [1]. It is an important concept and target for research and practice in health and medicine, and reflects individuals’ emotional, physical, material, and social wellbeing [1]. From April 2023, residents’ QoL was added as a reporting item in each quarter in the national Quality Indicator program [1].

Anticholinergic side effects may reduce users’ QoL. The potential association between anticholinergics and QoL has resulted in the adoption of QoL as an outcome measure in trials of anticholinergics deprescribing among older adults [5]. However, studies have reported mixed results on relationships between anticholinergic load and QoL [6–10]. Two main reasons for these mixed results are the use of different anticholinergic scales and not accounting for differences in study population characteristics, particularly dementia status. Various anticholinergic scales have been developed in different countries, e.g. Australia [11] Canada [12], and the USA [13–15]. Depending on distinct study purposes, higher scales scores were validated as related to higher serum anticholinergic activity [14], lower cognitive function [11], greater cognitive decline [12], and greater anticholinergic adverse effects [15]. Only two studies have been conducted in RACF populations. One study found that residents who used anticholinergics had lower psychological wellbeing than nonusers [9]. The other suggested that higher anticholinergic load seemed to be associated with lower QoL in residents without dementia, compared to higher QoL in those with dementia, but statistical testing was not conducted for either group [10].

We aimed to examine associations between anticholinergic load and QoL for residents in Australian RACFs and any differences between those with and without a dementia diagnosis.

## Methods

### Study design and setting

This was a retrospective cohort study using routinely collected data between 1 July and 31 December 2023, from 21 RACFs managed by a large not-for-profit aged care provider in New South Wales, Australia.

### Study population and data source

We included residents aged ≥65 years, who were permanent residents, had QoL assessments in 2023 at baseline (1 July–30 September) and at follow-up (1 October–31 December) in the same RACF.

Data for all included residents were collected from the aged care provider’s electronic information system, including year of birth, sex, entry and departure dates, health conditions recorded at entry to RACFs with updates when changes occurred during their stay, daily medication administration, and QoL assessments.

### Anticholinergic load and anticholinesterases

The Anatomical Therapeutic Chemical (ATC) classification codes were used to identify relevant medications. We applied five anticholinergic scales (Supplementary Table S1): Anticholinergic Cognitive Burden (ACB) [13], Anticholinergic Drug Scale (ADS) [14], Anticholinergic Loading Scale (ALS) [11], Anticholinergic Risk Scale (ARS) [15], and Clinician-rated Anticholinergic Score (CrAS) [12]. We chose these scales because they have been used in prior studies to examine relationships between anticholinergic load and QoL [6–10]. Anticholinergic load was calculated as the sum of anticholinergic ratings for different medications administered to a resident for the period of time between two QoL assessments. It was calculated using each of the five scales. Use of anticholinesterases (N06DA*) between two QoL assessments was coded as a binary variable.

### Quality of life

Quality of life was assessed using the Quality of Life Aged Care Consumers tool (QOL-ACC, Additional file 1) [16]. Consistent with the national program [1], QoL surveys were completed by residents, interviewer facilitated, or proxy-completion. The QOL-ACC comprises six statements for six attributes of QoL: mobility, pain management, emotional wellbeing, independence, social relationships, and leisure activities/hobbies. For instance, ‘I am able to get around as much as I want to (with the use of mobility aids e.g. wheelchair, walker, stick if you use them).’ There are five response options in a Likert scale to each statement: from ‘none of the time’ to ‘all of the time’. The total score can range from 0 to 24, with higher scores indicating better QoL. The study outcome was the follow-up QOL-ACC scores.

### Statistical analysis

For continuous variables with symmetrical distribution, means and standard deviations (SD) were reported. Medians and the first and third quartiles (Q1, Q3) were reported for continuous variables with skewed distributions. Frequencies and percentages were reported for categorical variables. Chi-squared tests were used to compare percentages of residents with versus those without dementia, who used anticholinergics as listed on any of the five scales under each ATC level 1 category.

Associations between anticholinergic load on each scale and follow-up QOL-ACC scores were determined using linear regression models. All models were adjusted for age, sex, baseline QOL-ACC score, the most recent record of health conditions, use of anticholinesterases, daily number of medications (excluding anticholinergics listed on the corresponding scale and anticholinesterases), QOL-ACC assessment mode at follow-up, days between two QoL assessments, years between the date of first entry to an RACF and that of the baseline QoL assessment. Health conditions considered in the current study (Table 1) are those related to both anticholinergic use and QoL [2, 3]. Facilities were adopted as a cluster variable.

**Table 1.** Characteristics of the study sample

	All residents (*n* = 927)	Residents with dementia (*n* = 426)	Residents without dementia (*n* = 501)
Age, mean ± SD	87.1 ± 7.7	86.5 ± 7.2	87.7 ± 8.0
Females, *n* (%)	646 (69.7)	313 (73.5)	333 (66.5)
Days between baseline and follow-up QOL-ACC, mean ± SD	95.8 ± 26.0	91.5 ± 24.8	99.4 ± 26.4
Anticholinergic load, median (Q1, Q3)			
Anticholinergic Cognitive Burden	1 (0, 2)	1 (0, 2)	1 (0, 2)
Anticholinergic Drug Scale	1 (0, 2)	1 (0, 1)	1 (0, 2)
Anticholinergic Loading Scale	1 (0, 2)	0 (0, 1)	1 (0, 2)
Anticholinergic Risk Scale	0 (0, 1)	0 (0, 1)	0 (0, 1)
Clinician-rated Anticholinergic Score	1 (0, 1)	0 (0, 1)	1 (0, 2)
Use of anticholinesterases	111 (12.0)	111 (26.1)	0 (0)
Daily number of medications not listed in the corresponding scale and not anticholinesterases, mean ± SD			
Anticholinergic Cognitive Burden	7.6 ± 3.5	6.8 ± 3.2	8.3 ± 3.6
Anticholinergic Drug Scale	7.5 ± 3.4	6.8 ± 3.1	8.1 ± 3.5
Anticholinergic Loading Scale	7.7 ± 3.5	6.9 ± 3.2	8.4 ± 3.6
Anticholinergic Risk Scale	7.9 ± 3.7	7.1 ± 3.3	8.7 ± 3.8
Clinician-rated Anticholinergic Score	7.8 ± 3.6	7.0 ± 3.3	8.5 ± 3.7
Years between the first entry to an RACF and baseline QOL-ACC, median (Q1, Q3)	1.7 (0.9, 3.8)	1.9 (1.0, 4.2)	1.4 (0.6, 3.4)
Health conditions, *n* (%)			
Dementia	426 (46.0)	-	-
Parkinson’s disease	70 (7.6)	36 (8.5)	34 (6.8)
Seizure	49 (5.3)	25 (5.9)	24 (4.8)
Schizophrenia or paranoid or psychotic states	71 (7.7)	46 (10.8)	25 (5.0)
Mood disorder^a^	468 (50.5)	235 (55.2)	233 (46.5)
Urinary incontinence	309 (33.3)	161 (37.8)	148 (29.5)
Chronic respiratory disease^b^	180 (19.4)	74 (17.4)	106 (21.2)
Gastroesophageal reflux disease or peptic ulcer	300 (32.4)	123 (28.9)	177 (35.3)
Stroke^c^	202 (21.8)	91 (21.4)	111 (22.2)
Cardiovascular disease^d^	809 (87.3)	363 (85.2)	446 (89.0)
Diabetes	236 (25.5)	92 (21.6)	144 (28.7)
QOL-ACC score			
Baseline, median (Q1, Q3)	22 (19, 24)	22 (19, 24)	22 (20, 24)
Follow-up, median (Q1, Q3)	22 (19, 24)	22 (19, 24)	22 (19, 23)
Follow-up QOL-ACC survey mode			
Self-completion	76 (8.2)	20 (4.7)	56 (11.2)
Interviewer facilitated completion	670 (72.3)	270 (63.4)	400 (79.8)
Proxy-completion	181 (19.5)	136 (31.9)	45 (9.0)

a Mood disorder includes depression, anxiety, bipolar disorder, posttraumatic stress disorder, or mood disorder that was not specified.

b Chronic respiratory disease includes chronic obstructive pulmonary disease and asthma.

c Stoke includes ischemic stroke, intracerebral haemorrhage, transient ischemic attack, and subarachnoid haemorrhage.

d Cardiovascular disease includes hypertension, cardiac arrhythmia, coronary heart disease, acute coronary syndrome, heart failure, and peripheral vascular disease.

We investigated medication administration between two QoL measurements, as recent anticholinergic load has been shown to be associated with greater risks of another important clinical endpoint, acute cardiovascular events [17]. Anticholinergic load was the most relevant within 30 days before cardiovascular events, compared to any other four 30-day reference periods between 61 and 180 days before events [17]. Second, the national quality indicator program is administered quarterly [1]. Relationships found for this time frame are likely to be meaningful for future trials on anticholinergic load reduction, as the matched assessment windows mean less measurement burden. However, in a sensitivity analysis, we extended investigated medication administration period. Time frames were from 91, 183, and 365 days prior to the baseline to the follow-up QoL measurement. Anticholinergic load, use of anticholinesterases, and daily number of medications were examined in these extended periods. Residents who newly entered RACF in the corresponding period were excluded from these analyses.

All analyses were conducted for overall cohort and a subgroup analysis was performed for residents with and without dementia. Anticholinesterases were not used in residents without dementia, and thus not adjusted for in this subgroup analysis. Interactions between anticholinergic load and dementia were tested. Assumptions of linearity, constant variance, and normality were checked by plotting the residuals against each of the explanatory variable and fitted values and by plotting histograms of residuals. Results are presented as β with 95% confidence interval (CI), with *P* < .05 regarded as statistically significant. Stata MP 18 (StataCorp LP, College Station, TX) was used.

## Results

**Figure 1:**
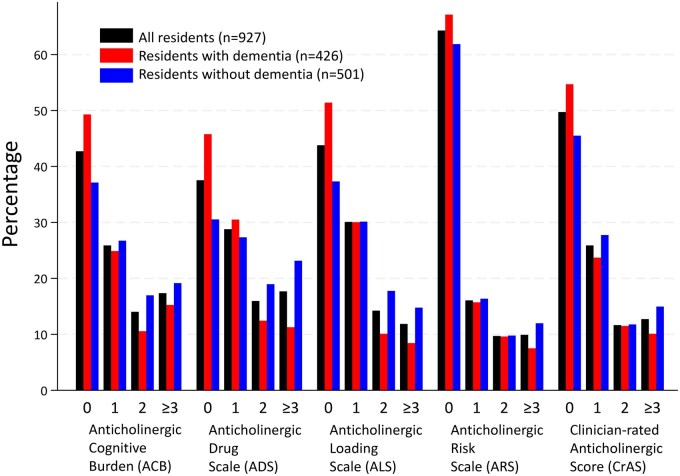
Percentages of residents with anticholinergic loads of 0, 1, 2, and ≥3

A total of 927 residents [646 females, 69.7%, mean age 87.1 years (SD 7.7)] were included in the analyses (Supplementary Fig. S1, Table 1). A larger proportion of residents without dementia received anticholinergics, compared to those with dementia. These included ATC level 1 categories: A alimentary tract and metabolism medications, C cardiovascular system, G genito urinary system and gender hormones, H systemic hormonal preparations excl. sex hormones and insulins, J anti-infectives for systemic use, and R respiratory system (Supplementary Table S2). Anticholinergics were used by 35.7% (ARS), 50.3% (CrAS), 56.2% (ALS), 57.3% (ACB), and 62.5% (ADS) of residents (Fig. 1, anticholinergic load of ≥1). The corresponding percentages were 32.9%, 45.3%, 48.6%, 50.7%, and 54.2% in residents with dementia, and 38.1%, 54.5%, 62.7%, 62.9%, and 69.5% in residents without dementia.

### Associations between anticholinergic load and QoL

**Figure 2:**
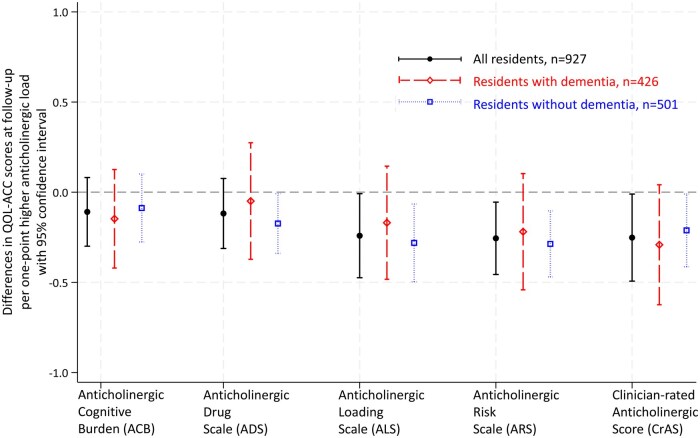
Associations between anticholinergic load and quality of life at follow-up. All models were adjusted for age, sex, baseline QOL-ACC score, the most recent record of health conditions, use of anticholinesterases (but not in the analyses for residents without dementia), daily number of medications excluding anticholinergics listed on the corresponding scale and anticholinesterases, QoL assessment mode at follow-up, days between baseline and follow-up QOL-ACC assessments, years between the date of first entry to a residential aged care facility and that of the baseline QOL-ACC assessment. Facility was adopted as a cluster variable

Linearity, constant variance, and normality held in all models. Among all residents, per one-point higher anticholinergic load measured on each of three scales was associated with lower QOL-ACC scores at follow-up: −0.24 (95% CI −0.47, −0.01) for ALS, −0.26 (95% CI −0.46, −0.05) for ARS, and −0.25 (95% CI −0.49, −0.01) for CrAS (Fig. 2, Supplementary Table S3). Associations between one-point higher anticholinergic load measured on the ACB and ADS and follow-up QoL were not statistically significant: −0.11 (95% CI −0.30, 0.08) and −0.12 (95% CI −0.31, 0.08), respectively.

### Residents with and without dementia

There were no differences in associations of anticholinergic load and QoL between residents with and without dementia (all *P* for the interaction >.05, Supplementary Table S3). In subgroup analyses, the associations remained statistically significant only among residents without dementia −0.28 (95% CI −0.50, −0.07) for ALS, −0.29 (95% CI −0.47, −0.10) for ARS, and −0.21 (95% CI −0.41, −0.01) for CrAS. Additionally, one-point higher anticholinergic load measured on ADS was related to lower QOL-ACC scores at follow-up among residents without dementia −0.17 (95% CI −0.34, −0.01).

### Sensitivity analyses

Excluding 71 residents (7.1% of the study sample) who newly entered the facility within 91 days before the baseline QoL measurements, the associations between anticholinergic load examined from 91 days before baseline through the end of the follow-up period and QoL were attenuated (Table S3). Similar attenuations were observed when anticholinergic load was examined from 183 and 365 days before baseline through the end of the follow-up period.

## Discussion

### Statement of principal findings

Controlling for QoL measured at baseline, a higher anticholinergic load assessed using the ALS, ARS, and CrAS was associated with a lower QoL at follow-up. The associations between anticholinergic load and QoL did not differ by dementia status. Yet, in a subgroup analysis for those with and without dementia, associations between higher anticholinergic load (assessed using each of the ADS, ALS, ARS, and CrAS) and lower QoL were statistically significant only in residents without dementia.

### Strength and limitations

The strength of the current study includes routinely collected daily medication administration data and QoL measured in a large sample using a validated tool designed for aged care recipients. We note a few limitations. First, residual confounding may present. People with health conditions such as urinary incontinence [18] have lower QoL compared to the general population. Thus, worse QoL could also be due to health conditions that are indications for anticholinergics. Although many of these health conditions were controlled for in our analyses, there may be inconsistencies in care practice regarding whether newly diagnosed conditions after entry to a RACF are updated in residents’ records. Second, although we adopted widely used anticholinergic scales, these scales and our data did not contain dose information. Anticholinergic burden from low- and high-dose use may have been overestimated and underestimated, respectively. Finally, we do not have information on who completed the QoL survey when it was proxy-completed, relatives or staff, but an earlier study suggested that proxy-ratings of QoL were similar between these two groups [19].

### Interpretation within the context of the wider literature

#### Anticholinergic scales

The five scales we adopted have been used to examine relationships between anticholinergic load and QoL [10–14]. Indeed, many more scales are available. Numbers of medications assessed as having anticholinergic effects vary. Between-scale differences in breadth of medication coverage were considerable [20, 21]. Out of a total of 543 medications from three scales (Chew’s list, ADS, and ARS), only 25 were listed in all [20]. While 78% of medications in the ACB were also in the ADS, conversely only 16% of the ADS medications were also in the ACB [21]. This discrepancy affects prevalence estimations. Similar to the wide range of 35.7%–62.5% residents who used any anticholinergics we observed, prior studies reported ranges of 22.8%–55.9% (nine scales, 1-year prevalence in those aged ≥65 years) [22] and 8%–17.6% (10 scales, point prevalence in those aged 37–73 years) [23]. Ratings of anticholinergic effects for the same medications also varied between scales. Spearman’s correlation coefficient ranged from 0.12 to 0.82 between assigned scores of overlapping medications [21]. For example, in the current work, anticholinergic activity of loperamide, an antidiarrheal, is suggested as moderate (score of 2) in the ARS, compared to as mild (score of 1) in other four scales.

#### Associations between anticholinergic load and QoL

Variations in measurements and study populations are considerable in longitudinal studies examining associations between anticholinergic load and QoL [4]. QoL has been evaluated using a range of instruments including the Short-Form 12 [6] and 36 [7] surveys, EuroQoL five dimensions three-level version [24], and McGill Quality of Life Scale [8]; and used as continuous [6, 7, 24] or ordinal outcomes in previous studies [8]. Anticholinergic load and QoL have been repeatedly measured (four yearly [7] and three half-yearly [24]) in two community samples and for seven periods prior to death in a palliative care population [8]. In a community sample of older adults with dementia, after baseline measures of QoL, new users of medications with moderate to high anticholinergic effects assessed on the ADS had 7.48 points lower health-related QoL at follow-up, compared to nonusers [6].

Despite many heterogeneities, the effect size of 7.48 out of a 100-point scale (0.8%) was similar to our finding of ∼0.25 out of a 30-point scale (0.8%). The initial implementation process may mean inconsistencies in how staff administered QoL measurements (internal communications), and thus, we used the second QoL assessment period after when this quality indicator was required at national level as the baseline [1]. We cannot comment on the clinical meaningfulness of the 0.25-point differences, but these were in the context of no substantial differences in QoL during the two assessments periods we investigated (proportions of RACF residents who scored ≥19 on the QOL-ACC 72.5% versus 72.6%) [25].

Cross-sectional data in 2011 [9] and 2017 [10] were collected from all older adults aged ≥65 years who lived in Helsinki ‘nursing homes’ and ‘assisted living facilities’. QoL was assessed using the World Health Organization Quality of Life-Brief [9] and 15D [10]. Better QoL was found among those who did not use any anticholinergics on the ARS, compared to gradually lower QoL among those who used one, two, to ≥3 anticholinergics (*P* for trend <0.001 adjusting for age and sex) [9]. Inversely, an association between higher ARS anticholinergic load and better QoL was found [10]. Mixed findings in prior studies, longitudinal and cross-sectional, suggest relationships and strengths of relationships differ depending on case-mix and how anticholinergics and QoL were measured and examined (e.g. variations in scales, measures, measurement intervals, and analytical methods).

Such variations were minimized in the current study [4, 6–10, 24], as we, for the first time, examined the relationship between anticholinergics and QoL in the same study population of old adults in RACFs and used the same scale for QoL and analytical methods. Thus, variations in our findings mainly resulted from differences among the five anticholinergic scales, such as the anticholinergics included and how they were scored. Although these ratings were made primarily based on medications’ effects on cognitive function, our findings underscore the utility of the ALS, ARS, and CrAS in evaluating QoL.

#### Residents with and without dementia

In a prior study of RACF residents, opposite directions of associations between anticholinergic load and QoL were found between residents with and without dementia (*P* for interaction 0.021), adjusting for age, sex, and Charlson Comorbidity Index [10]. However, our finding of no effect modification by dementia status may be more plausible due to two important adjustments. First, we controlled for the baseline QOL-ACC, compared to the cross-sectional nature of the prior study. Second, our earlier work found that proxy-completed and interviewer facilitated QOL-ACC scores are lower than self-completed QoL surveys [26]. However, although three assessment modes for QoL were also adopted in the prior study, the mode was not controlled for [10].

We observed wide CIs in residents with dementia, which could explain the nonsignificant associations between anticholinergics and QoL in this subgroup. There are two possible reasons for the wide CIs. First, the percentages of anticholinergic users were lower in residents with dementia: 32.9%–54.2% (depending on scales) versus those without dementia: 38.1%–69.5%. Additionally, some categories of anticholinergics (ATC level 1 A, C, G, H, J, and R) were used by statistically significantly smaller proportions of residents with dementia than those without dementia. This may be due to more frequent referencing of anticholinergic risks in guidelines for older adults with dementia [2]. Consistent with our findings, data of 4478 residents in Scottish RACF showed that residents with dementia were 39% less likely to be prescribed anticholinergics than those without dementia [27], although others showed no differences in proportions of residents with and without cognitive impairment who used strong anticholinergics [28]. Second, the QOL-ACC was completed by proxy in 31.9% of those with dementia compared to 9% of those without dementia. There are potentially greater variations within proxy-completed QoL than within self-completed or interviewer facilitated ones, because proxies’ perspectives could make a difference. For example, their evaluations of QoL differed depending on whether they were asked to assess QoL from the patient’s point of view or based on their own perceptions of the patient’s QoL [29]. Accordingly, attenuation of associations in sensitivity analyses may be due to increased proportion of residents with dementia in these analyses. However, it may also be due to the reduced sample size and the fact that recent anticholinergic effect is more relevant than load in a prolonged period [17].

### Implications for policy, practice, and research

Medication-related issues and residents’ QoL in residential aged care are both highlighted in Australian national quality improvement programs. Given the increased availability of electronic medication systems in RACFs, we recommend the inclusion of anticholinergic scales be incorporated, to automatically identify residents who may need more support for their wellbeing. Due to regional variations in guidelines, prescribing habits, and availability of medications, the best choice of anticholinergic scale may differ between populations. Further research is required to support the selection of appropriate anticholinergic scales incorporating dose information.

## Conclusions

Use of anticholinergics was associated with lower QoL in an Australian RACF population. However, we found associations varied depending on the adopted anticholinergic scale, suggesting that some scales have identified and rated medications in a way that is more relevant to QoL. The identified relationships suggest that anticholinergics deprescribing may improve residents’ QoL.

## Supplementary Material

mzaf123_Supplementary_Data

## Data Availability

The data for this study are not publicly available. All authors have full access to the data, but do not have permission from the residents or ethics approval to share the data.
