# Supplementary material for: Anticholinergic load and quality of life in Australian residential aged care: a retrospective cohort study
Source: Int J Qual Health Care. 2025 Dec 16;38(1):mzaf123. doi: 10.1093/intqhc/mzaf123 (PMC12817077; doi:10.1093/intqhc/mzaf123)
Supplement: mzaf123_Supplementary_Data [file mzaf123_supplementary_data.zip › Supplementary.pdf]

Supplementary

This supplementary file is to provide additional information for article: Anticholinergic load and quality of life in Australian residential aged care: a retrospective cohort study

Table of Contents

Additional file 1 Quality of Life Aged Care Consumers *© Flinders University 2022* 1

Figure S1 Flow chart of analysed residents 2

Table S1 Five anticholinergic scales used in the study 3

Table S2 Administered medications in all residents, residents with and without dementia and by scale 4

Table S3 Associations between anticholinergic load and quality of life at follow-up 7

Additional file 1 Quality of Life Aged Care Consumers © *Flinders University 2022*

There are six dimensions and statements in this tool:

1. Mobility: I am able to get around as much as I want to (with the use of mobility aids e.g., wheelchair, walker, stick if you use them).
2. Pain management: When I experience pain, it is well managed.
3. Emotional wellbeing: I am generally happy.
4. Independence: I have as much independence as I want.
5. Social relationships: I have good social relationships with family and friends;
6. Leisure activities/hobbies: I have leisure activities/hobbies I enjoy.

Each question has five response options (corresponding score):

1. All of the time (score of 4)
2. Most of the time (score of 3)
3. Some of the time (score of 2)
4. A little of the time (score of 1)
5. None of the time (score of 0)

More details: https://www.qol-acc.org/quality-of-life-instrument or Appendix D of https://www.health.gov.au/sites/default/files/2023-11/national-aged-care-mandatory-quality-indicator-program-manual-3-0-part-a.pdf

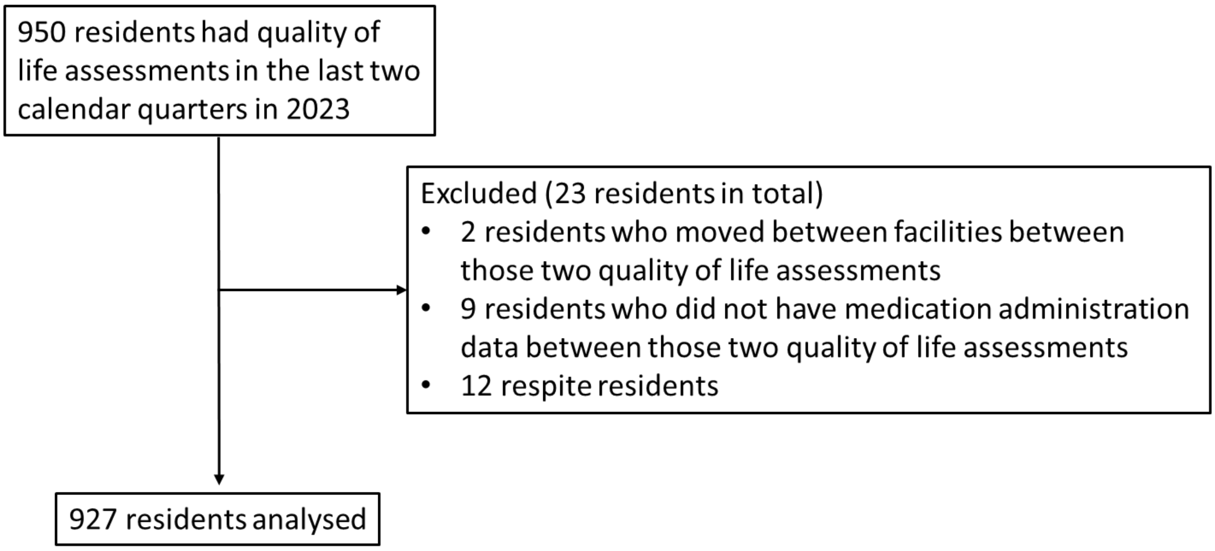

Figure S1 Flow chart of analysed residents

Table S1 Five anticholinergic scales used in the study

Scale, country, ratings, year, first author	Details on studies where the scale was developed	
	Study sample	Key findings
Anticholinergic Loading Scale<br>Australia<br>0, 1, 2, 3<br>2011<br>Sittironnarit	The Australian Imaging, Biomarkers and Lifestyle study of ageing: cohort of individuals aged ≥60 years. Baseline data were used.	Higher anticholinergic load was cross-sectionally associated with lower psychomotor speed and executive function, but not on other areas of cognition in cognitively healthy older adults. It was not associated with cognition in participants with mild cognitive impairment or Alzheimer's disease.
Anticholinergic Cognitive Burden<br>USA<br>1, 2, 3<br>2008<br>Boustani	A Medline search identified studies evaluating anticholinergic use and relationships between anticholinergics and cognitive impairment.	In this review, the authors suggested to consider alternative medications with ACB <3 and reduce a total ACB score to <3.
Anticholinergic Drug Scale<br>USA<br>0, 1, 2, 3<br>2006<br>Carnahan	Residents in rural long-term care facilities (n=201, aged 64 to 102 years).	Higher ADS load was associated with higher serum anticholinergic activity. A 1:2:3 proportion for the scores on the ADS is reasonable.
Anticholinergic Risk Scale<br>USA<br>1, 2, 3<br>2008<br>Rudolph	A retrospective cohort of 132 participants seen consecutively in geriatric evaluation and management clinics at the Veterans Affairs Boston Healthcare System and a prospective cohort of 117 male participants from a primary care cohort, all aged ≥65 years.	Higher ARS load was associated with greater rates of having central (any of falls, dizziness, and confusion) and peripheral anticholinergic adverse effects (any of dry mouth, dry eyes, and constipation).
Clinician-rated Anticholinergic Score<br>Canada<br>0, 1, 2, 3<br>2008<br>Han	Connecticut Veterans Longitudinal Cohort: 767 veterans aged ≥65 years recruited at a Department of Veterans Affairs primary care clinic.	Higher cumulative exposure to anticholinergic medications over one year was associated with lower memory and executive function, adjusting for baseline memory and executive function and other covariates.

Table S2 Administered medications in all residents, residents with and without dementia and by scale

ATC code, generic name, by ATC level 1 category*	n (%)			Scores by scale				
	All residents (n=927)	Dementia						
		Yes (n=426)	No (n=501)	ACB	ADS	ALS	ARS	CrAS
A02BA03 famotidine	4 (0.4)	1 (0.2)	3 (0.6)		1			
A02BA04 nizatidine	12 (1.3)	1 (0.2)	11 (2.2)		1			
A03BA01 atropine	1 (0.1)	1 (0.2)	0 (0)	3	3	3	3	3
A03FA01 metoclopramide	32 (3.5)	7 (1.6)	25 (5.0)			1	1	3
A03FA03 domperidone	22 (2.4)	8 (1.9)	14 (2.8)			1		
A06AB02 bisacodyl	19 (2.0)	7 (1.6)	12 (2.4)			1		
A07DA03 loperamide	20 (2.2)	5 (1.2)	15 (3.0)	1	1	1	2	1
A10BA02 metformin	103 (11.1)	37 (8.7)	66 (13.2)			1		
**A alimentary tract and metabolism***	**189 (20.4)**	**60 (14.1)**	**129 (25.7)**					
B01AA03 warfarin	26 (2.8)	8 (1.9)	18 (3.6)	1	1			
**B blood and blood forming organs**	**26 (2.8)**	**8 (1.9)**	**18 (3.6)**					
C01AA05 digoxin	70 (7.6)	21 (4.9)	49 (9.8)	1	1	1		
C01DA14 isosorbide mononitrate	29 (3.1)	12 (2.8)	17 (3.4)	1	1			
C02DB02 hydralazine	7 (0.8)	0 (0)	7 (1.4)	1	1			
C03CA01 furosemide	256 (27.6)	90 (21.1)	166 (33.1)	1	1			
C07AB02 metoprolol	132 (14.2)	49 (11.5)	83 (16.6)	1				1
C07AB03 atenolol	34 (3.7)	16 (3.8)	18 (3.6)	1				1
C08CA05 nifedipine	2 (0.2)	0 (0)	2 (0.4)	1	1			
C08DB01 diltiazem	4 (0.4)	0 (0)	4 (0.8)		1			
C09AA10 trandolapril	2 (0.2)	2 (0.5)	0 (0)					1
**C cardiovascular system***	**397 (42.8)**	**151 (35.4)**	**246 (49.1)**					
G04BD04 oxybutynin	18 (1.9)	4 (0.9)	14 (2.8)	3	3	2	3	
**G genito urinary system and gender hormones***	**18 (1.9)**	**4 (0.9)**	**14 (2.8)**					
H02AB02 dexamethasone	2 (0.2)	0 (0)	2 (0.4)		1			
H02AB06 prednisolone	43 (4.6)	14 (3.3)	29 (5.8)		1			
H02AB07 prednisone	41 (4.4)	13 (3.1)	28 (5.6)	1	1			
H02AB10 cortisone	3 (0.3)	1 (0.2)	2 (0.4)		1			
**H systemic hormonal preparations, excl. gender hormones and insulins***	**85 (9.2)**	**26 (6.1)**	**59 (11.8)**					
J01FF01 clindamycin	20 (2.2)	4 (0.9)	16 (3.2)		1			

ATC code, generic name, by ATC level 1 category*	n (%)			Scores by scale				
	All residents (n=927)	Dementia						
		Yes (n=426)	No (n=501)	ACB	ADS	ALS	ARS	CrAS
**J anti-infectives for systemic use***	**20 (2.2)**	**4 (0.9)**	**16 (3.2)**					
L04AD01 ciclosporin	1 (0.1)	1 (0.2)	0 (0)		1			
L04AX03 methotrexate	8 (0.9)	4 (0.9)	4 (0.8)			1		
**L antineoplastic and immunomodulating agents**	**9 (1.0)**	**5 (1.2)**	**4 (0.8)**					
M01AH01 celecoxib	3 (0.3)	2 (0.5)	1 (0.2)			1		
M03BX01 baclofen	4 (0.4)	1 (0.2)	3 (0.6)				2	2
M04AC01 colchicine	5 (0.5)	0 (0)	5 (1.0)	1				
**M musculo-skeletal system**	**12 (1.3)**	**3 (0.7)**	**9 (1.8)**					
N02AA01 morphine	26 (2.8)	9 (2.1)	17 (3.4)	1	1			1
N02AA05 oxycodone	112 (12.1)	39 (9.2)	73 (14.6)		1	1		1
N02AB03 fentanyl	5 (0.5)	3 (0.7)	2 (0.4)	1	1			
N02AJ06 codeine	12 (1.3)	7 (1.6)	5 (1.0)	2	1	1		1
N02AX02 tramadol	8 (0.9)	0 (0)	8 (1.6)		1	2		2
N03AE01 clonazepam	16 (1.7)	7 (1.6)	9 (1.8)		1	1		
N03AF01 carbamazepine	11 (1.2)	7 (1.6)	4 (0.8)	2	2			1
N03AG01 valproic acid	39 (4.2)	28 (6.6)	11 (2.2)		1			
N04AC01 benzatropine	3 (0.3)	1 (0.2)	2 (0.4)	3	3		3	
N04BB01 amantadine	1 (0.1)	0 (0)	1 (0.2)	2	1		2	
N04BC05 pramipexole	22 (2.4)	6 (1.4)	16 (3.2)				1	
N04BX02 entacapone	1 (0.1)	0 (0)	1 (0.2)				1	
N05AB04 prochlorperazine	12 (1.3)	6 (1.4)	6 (1.2)		1	2	2	2
N05AD01 haloperidol	2 (0.2)	1 (0.2)	1 (0.2)	1		2	1	
N05AH02 clozapine	2 (0.2)	2 (0.5)	0 (0)	3	3		2	
N05AH03 olanzapine	17 (1.8)	6 (1.4)	11 (2.2)	3	1		2	1
N05AH04 quetiapine	40 (4.3)	28 (6.6)	12 (2.4)	3			1	2
N05AN01 lithium	4 (0.4)	1 (0.2)	3 (0.6)			1		
N05AX08 risperidone	29 (3.1)	22 (5.2)	7 (1.4)	1		1	1	1
N05BA01 diazepam	14 (1.5)	3 (0.7)	11 (2.2)	1	1	1		1
N05BA04 oxazepam	4 (0.4)	0 (0)	4 (0.8)		1	1		
N05BA06 lorazepam	21 (2.3)	9 (2.1)	12 (2.4)		1			
N05BA12 alprazolam	1 (0.1)	0 (0)	1 (0.2)	1	1	1		1
N05CD07 temazepam	51 (5.5)	12 (2.8)	39 (7.8)		1	1		
N05CD08 midazolam	9 (1.0)	6 (1.4)	3 (0.6)		1			
N06AA04 clomipramine	2 (0.2)	1 (0.2)	1 (0.2)	3	3			
N06AA09 amitriptyline	23 (2.5)	8 (1.9)	15 (3.0)	3	3	3	3	3
N06AA10 nortriptyline	3 (0.3)	1 (0.2)	2 (0.4)	3	3		2	3
N06AA12 doxepin	1 (0.1)	0 (0)	1 (0.2)	3	3	3		3
N06AA16 dosulepin	1 (0.1)	1 (0.2)	0 (0)			2		

ATC code, generic name, by ATC level 1 category*	n (%)			Scores by scale				
	All residents (n=927)	Dementia						
		Yes (n=426)	No (n=501)	ACB	ADS	ALS	ARS	CrAS
N06AB03 fluoxetine	6 (0.6)	3 (0.7)	3 (0.6)		1	1		1
N06AB04 citalopram	65 (7.0)	39 (9.2)	26 (5.2)			1		
N06AB05 paroxetine	4 (0.4)	3 (0.7)	1 (0.2)	3	1	2	1	2
N06AB06 sertraline	52 (5.6)	30 (7.0)	22 (4.4)		1			1
N06AB08 fluvoxamine	3 (0.3)	1 (0.2)	2 (0.4)	1	1	1		
N06AB10 escitalopram	47 (5.1)	23 (5.4)	24 (4.8)			1		
N06AX11 mirtazapine	149 (16.1)	75 (17.6)	74 (14.8)				1	
N06AX16 venlafaxine	39 (4.2)	15 (3.5)	24 (4.8)			1		1
**N nervous system**	**521 (56.2)**	**236 (55.4)**	**285 (56.9)**					
R01BA02 pseudoephedrine	1 (0.1)	0 (0)	1 (0.2)			2	2	
R03AK06 salmeterol and fluticasone	55 (5.9)	14 (3.3)	41 (8.2)		1			
R05DA04 codeine	2 (0.2)	0 (0)	2 (0.4)	1	1	1		1
R06AD02 promethazine	5 (0.5)	2 (0.5)	3 (0.6)	3	3		3	
R06AE07 cetirizine	10 (1.1)	4 (0.9)	6 (1.2)			2	2	2
R06AX02 cyproheptadine	1 (0.1)	0 (0)	1 (0.2)	2	2	3	3	
R06AX13 loratadine	42 (4.5)	12 (2.8)	30 (6.0)			1	2	1
R06AX26 fexofenadine	14 (1.5)	4 (0.9)	10 (2.0)			2		2
**R respiratory system***	**121 (13.1)**	**35 (8.2)**	**86 (17.2)**					
S01GX08 ketotifen	1 (0.1)	0 (0)	1 (0.2)		1			
**S sensory organs**	**1 (0.1)**	**0 (0)**	**1 (0.2)**					

ACB denotes Anticholinergic Cognitive Burden, ADS Anticholinergic Drug Scale, ALS Anticholinergic Loading Scale, ARS Anticholinergic Risk Scale, ATC code Anatomical Therapeutic Chemical classification code, and CrAS Clinician-rated Anticholinergic Score.

*Lower percentages of residents with dementia used anticholinergics listed on any of the five scales, under this ATC level 1 category, than those without dementia.

Table S3 Associations between anticholinergic load and quality of life at follow-up

	Scale	β (95% confidence interval)	*p* value	β (95% confidence interval)		*p* value	*p* value interaction by dementia
Main analyses (n=927, 426 with dementia, 501 without dementia)	ACB	-0.11 (-0.30, 0.08)	0.25	Dementia	-0.15 (-0.42, 0.13)	0.27	0.95
				No dementia	-0.09 (-0.28, 0.10)	0.34	
	ADS	-0.12 (-0.31, 0.08)	0.22	Dementia	-0.05 (-0.37, 0.27)	0.75	0.09
				No dementia	**-0.17 (-0.34, -0.01)**	**0.04**	
	ALS	**-0.24 (-0.47, -0.01)**	**0.04**	Dementia	-0.17 (-0.48, 0.14)	0.27	0.22
				No dementia	**-0.28 (-0.50, -0.07)**	**0.01**	
	ARS	**-0.26 (-0.46, -0.05)**	**0.02**	Dementia	-0.22 (-0.54, 0.10)	0.17	0.37
				No dementia	**-0.29 (-0.47, -0.10)**	**0.004**	
	CrAS	**-0.25 (-0.49, -0.01)**	**0.04**	Dementia	-0.29 (-0.62, 0.04)	0.08	0.85
				No dementia	**-0.21 (-0.41, -0.01)**	**0.04**	
91 days prior to baseline (n=856, 403 with dementia, 453 without dementia)	ACB	-0.09 (-0.28, 0.10)	0.34	Dementia	-0.12 (-0.39, 0.15)	0.37	0.94
				No dementia	-0.08 (-0.26, 0.09)	0.34	
	ADS	-0.09 (-0.27, 0.09)	0.30	Dementia	-0.07 (-0.41, 0.26)	0.66	0.33
				No dementia	-0.12 (-0.28, 0.04)	0.13	
	ALS	-0.17 (-0.37, 0.03)	0.10	Dementia	-0.13 (-0.44, 0.18)	0.38	0.42
				No dementia	**-0.18 (-0.34, -0.02)**	**0.03**	
	ARS	-0.15 (-0.32, 0.01)	0.07	Dementia	-0.17 (-0.46, 0.12)	0.24	0.74
				No dementia	**-0.16 (-0.31, -0.01)**	**0.04**	
	CrAS	-0.17 (-0.36, 0.02)	0.07	Dementia	-0.20 (-0.48, 0.08)	0.15	0.99
				No dementia	-0.14 (-0.30, 0.01)	0.07	
183 days prior to baseline (n=769, 376 with dementia, 393 without dementia)	ACB	-0.11 (-0.31, 0.09)	0.26	Dementia	-0.17 (-0.47, 0.13)	0.25	0.75
				No dementia	-0.07 (-0.24, 0.10)	0.38	
	ADS	-0.08 (-0.23, 0.07)	0.28	Dementia	-0.10 (-0.40, 0.20)	0.48	0.53
				No dementia	-0.08 (-0.21, 0.05)	0.21	
	ALS	-0.13 (-0.32, 0.07)	0.18	Dementia	-0.14 (-0.51, 0.22)	0.43	0.87
				No dementia	-0.10 (-0.23, 0.03)	0.11	
	ARS	-0.15 (-0.33, 0.03)	0.10	Dementia	-0.15 (-0.45, 0.15)	0.31	0.59
				No dementia	-0.17 (-0.37, 0.03)	0.10	
	CrAS	**-0.17 (-0.34, -0.001)**	**0.05**	Dementia	-0.20 (-0.50, 0.09)	0.17	0.90
				No dementia	**-0.13 (-0.24, -0.02)**	**0.02**	
365 days prior to baseline (n=648, 331 with dementia, 317 without dementia)	ACB	-0.15 (-0.36, 0.07)	0.17	Dementia	-0.19 (-0.48, 0.09)	0.16	0.84
				No dementia	-0.11 (-0.31, 0.10)	0.29	
	ADS	-0.12 (-0.28, 0.05)	0.15	Dementia	-0.12 (-0.37, 0.13)	0.33	0.55
				No dementia	-0.13 (-0.31, 0.05)	0.15	
	ALS	-0.14 (-0.35, 0.08)	0.21	Dementia	-0.12 (-0.43, 0.18)	0.40	0.84
				No dementia	-0.14 (-0.33, 0.06)	0.15	
	ARS	-0.16 (-0.37, 0.05)	0.14	Dementia	-0.18 (-0.48, 0.11)	0.21	0.94
				No dementia	-0.14 (-0.37, 0.09)	0.23	
	CrAS	-0.18 (-0.40, 0.03)	0.09	Dementia	-0.22 (-0.55, 0.11)	0.18	0.72
				No dementia	-0.13 (-0.29, 0.04)	0.12	

ACB denotes Anticholinergic Cognitive Burden, ADS Anticholinergic Drug Scale, ALS Anticholinergic Loading Scale, ARS Anticholinergic Risk Scale, and CrAS Clinician-rated Anticholinergic Score.

β indicates differences in QOL-ACC scores at follow-up per one-point higher anticholinergic load.

All models were adjusted for age, sex, baseline QOL-ACC score, the most recent record of health conditions, use of anticholinesterases (but not in the analyses for residents without dementia), daily number of medications excluding anticholinergics listed on the corresponding scale and anticholinesterases, QoL assessment mode at follow-up, days between baseline and follow-up QOL-ACC assessments, years between the date of first entry to a residential aged care facility and that of the baseline QOL-ACC assessment. Facility was adopted as a cluster variable.
